# Environmental impact ratings that could drive positive environmental changes in the manufacture and use of pharmaceuticals

**DOI:** 10.3399/BJGPO.2021.0214

**Published:** 2022-01-12

**Authors:** Amelia Cussans, Guy Harvey, Terry Kemple, Tracy Lyons, Mike Tomson, Angela Wilson

**Affiliations:** 1 Psychiatry Trainee, Central North West London NHS Trust Mental Health Centre, London, UK; 2 Member of the RCPsych Planetary Health and Sustainability Committee. Consultant Psychiatrist, Cumbria Northumberland Tyne and Wear NHS Trust, Newcastle upon Tyne, UK; 3 Royal College of General Practitioners National Representative for Sustainability, Climate Change and Green Issues, Lead for Green Impact for Health Toolkit, Executive member of UK Health Alliance for Climate Change, London, UK; 4 Medicines Optimisation Pharmacist, Directorate Sustainability Lead, Radiology & Pharmacy, University Hospital Dorset NHS Foundation Trust, Poole, UK; 5 Retired GP, Sheffield, UK; 6 Locum, Oxfordshire Clinical Commissioning Group, UK

**Keywords:** environment, Quality assurance, pharmaceuticals, general practice, primary healthcare

The climate and ecological emergency (CEE) is the greatest health threat of the 21^st^ century.^1^ Many clinicians work hard to reduce their personal carbon footprint. This contrasts with our work lives, where we have limited scope to reduce the environmental impact of the care we provide. Our priorities lie in delivering the best possible care to the patients in front of us, so this dissonance often goes unaddressed. Yet paradoxically our services may contribute to climate-related health hazards. We will all feel the impacts of the CEE, but the health burden will impact most on future generations ^2^ and low-income, minority and politically-marginalised groups.^3^ This is a matter of global and intergenerational justice. All industries have a moral obligation ^4^ to reduce their carbon emissions, and as an organisation dedicated to protecting public health, the NHS has an imperative to do so.

The pharmaceutical industry plays a central role in health care, yet it is also an area of growing concern. Globally, its emissions intensity is 55 % higher than the automotive sector.^5^ The supply chain ^6^ — pharmaceuticals and medical instruments — contributes the highest proportion of the NHS’s greenhouse gas (GHG) emissions. Given that the NHS produces 4 % of the UK’s total GHG emissions,^6^ pharmaceuticals are a major contributor to the climate crisis. Indeed, in primary care, medicines have been identified as a ‘carbon hotspot’, accounting for 65 % of its total GHG emissions.^7^

In addition to their global warming potential, human and veterinary pharmaceuticals have wide-ranging environmental impacts. They are released into natural systems during manufacture, use, and disposal, even following excretion by the patient. Drugs accumulate in water sources where they can have toxic effects on aquatic ecosystems.^8^ The body of evidence for this is mounting. Non-steroidal anti-inflammatory drugs (NSAIDs) have been found in otter fur.^9^ Hormonal contraceptives disrupt frog fertility.^10^ Venlafaxine can cause freshwater snails ^11^ to detach from surfaces. Citalopram reduces predator avoidance behaviours in crayfish.^12^ These drugs enter the food chain and ultimately make their way back to humans; the health impacts of this remain unknown.

Based on ‘first do no harm‘ it is essential that we act quickly and collectively to mitigate the environmental impact of pharmaceuticals. Our General Practitioners, Psychiatrists and Pharmacists Working Group has focused on how we can reduce the environmental impact of pharmaceuticals from research, manufacture, packaging, and sterilisation, through to transport, consumption, elimination, and disposal. Improving our prescribing practices through medicines optimisation is key to making pharmaceuticals more sustainable, although this is beyond our scope here.

We know there is great variation in drug companies’ environmental performance. All other things being equal, clinicians and patients prefer to use the least polluting medications. But it would be hard for clinicians to factor in the green credentials of each pharmaceutical to every prescribing decision. Nor would it be feasible to undertake carbon calculation for each individual drug. Instead, the NHS could categorise manufacturers based on their progress towards environmental commitments. Drugs could then be procured preferentially from companies with the best green performance.

Our working group proposes an Environmental Impact Rating (EIR) system for NHS pharmaceutical manufacturers, based on the environmental impact of their products. The EIR would consider the entire pharmaceutical lifecycle. This covers manufacture, sterilisation, packaging, and waste products. Stages that take place outside of the UK must be accounted for. All four of the UK nations have committed as part of the UK COP26 Presidency’s Health Programme to become net zero health services. The NHS England (NHSE) net zero plan commits to carbon neutrality by 2040 for direct and by 2045 for indirect emissions, which includes the supply chain. In the NHSE plan, pharmaceutical companies will have to match these environmental commitments to continue to supply the NHSE with drugs after 2030. We believe in an earlier net zero target in line with the current climate science,^13^ which states that we need to be more ambitious ^14^ to mitigate the worst effects of climate change. The EIR rating asks for data on ecotoxicity as well as GHG emissions because a drug could theoretically be carbon-neutral yet still a potent eco-toxin.

**Figure 1.**
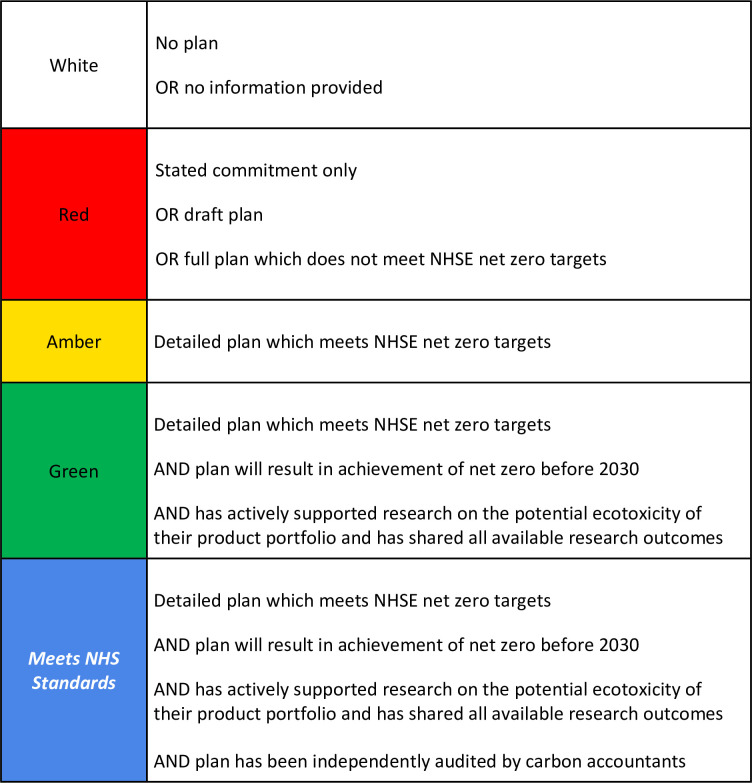
NHS England manufacturer Environmental Impact Rating (EIR) status categories

We propose a traffic light EIR rating system with categories that could look like Figure 1.

The NHSE should reward manufacturers with the least environmental impact by preferentially purchasing their products. Any excess cost could be limited. For example, if the acceptable price difference between each of the 5 EIR rankings was set at 10% of the cost of the cheapest product, then the cheapest product from the manufacturer with the worst environmental impact could be purchased if the product from the manufacturer with the least environmental impact cost 40% more than the cheapest.This system would create a competitive advantage for companies who achieve zero carbon early, actively encouraging them to improve their environmental performance. Ultimately, we want all manufacturers to achieve these standards in all UK nations as soon as possible. This would accelerate the NHS’s journey towards net zero.^6^

Manufacturers should publish transparent and detailed plans, including calculations of current and projected carbon emissions. Their plans should be accompanied by a separate report from independent carbon auditors. This should include a robustly measured carbon footprint and comments on the strength and credibility of the manufacturer’s plan. This would reduce the risk of greenwashing. There are a number of carbon accounting services available and these services are expected to grow as demand increases. The NHSE would need to establish which methodology can reliably be used so that reports are comparable. Manufacturers’ compliance with their plans should be subject to annual audits to calculate current footprints and to ensure they are meeting their targets.

An area that requires further consideration is how this system should appraise and factor in the impact of specific products. For example, Salamol inhalers contain half as much hydrofluorocarbon propellant ^15^ as Ventolin inhalers for an equivalent dosage, halving their carbon footprint. Data of this nature will need to be considered as well as the green credentials at a company level.

In time, we hope meeting these standards will be tied to legislative requirements. It is worth noting that legal experts worldwide are seeking to make ‘ ecocide’ an international crime ^16^ within the jurisdiction of the International Criminal Court. A definition of ecocide has recently been unveiled: *’unlawful or wanton acts committed with knowledge that there is a substantial likelihood of severe and widespread or long-term damage to the environment being caused by those acts*‘. If adopted by the International Criminal Court then corporations, including those manufacturing pharmaceuticals, could be prosecuted for serious offences against the environment. We hope this may be a catalyst for faster change.

The *Delivering a ‘* *Net Zero’* *National Health Service* ^6^ report states ‘*the NHS has a responsibility to use its purchasing power to drive positive environmental, social and economic change and add value for the communities it serves* *’*. Our Working Group believes the EIR is a viable intervention which would allow doctors and patients to be confident that the medicines they use are from the greenest manufacturers.
